# Osteoporosis and CKD-Metabolic Bone Disease Under the Same Umbrella: Insights From a Joint Scientific Symposium

**DOI:** 10.1016/j.ekir.2026.106362

**Published:** 2026-02-17

**Authors:** David W. Dempster, Pieter Evenepoel, Thomas L. Nickolas, Ziad A. Massy, Sandro Mazzaferro, Nicholas C. Harvey, Paul D. Miller, Michael Pazianas

**Affiliations:** 1 Vagelos College of Physicians and Surgeons of Columbia University, New York, New York, USA; 2 Department of Microbiology, Immunology, and Transplantation, Laboratory of Nephrology, KU Leuven, Herestraat, Leuven, Belgium; 3 Division of Bone and Mineral Diseases, Department of Medicine, Washington University School of Medicine, St. Louis, Missouri, USA; 4 Association pour L'utilisation du rein Artificiel en Région Parisienne, Paris, France; 5 Inserm Unit 1018, Team 5, CESP, Hôpital Paul Brousse, Paris-Saclay University and Versailles Saint-Quentin-en-Yvelines University, Villejuif, France; 6 Department of Nephrology, Ambroise Paré University Hospital, APHP, Paris, France; 7 Department of Translation and Precision Medicine, Sapienza University of Rome, Rome, Italy; 8 MRC Lifecourse Epidemiology Centre, University of Southampton, Southampton, UK; 9 NIHR Southampton Biomedical Research Centre, University of Southampton and University Hospital Southampton NHS Foundation Trust, Southampton, UK; 10 University of Colorado Health Sciences Center, Denver, Colorado, USA; 11 Colorado Center for Bone Health, Lakewood, Colorado, USA; 12 Institute for Translational Medicine and Pharmacology, Icahn Sinai School of Medicine at Mount Sinai, New York, New York, USA

**Keywords:** CKD-MBD, osteoporosis, renal osteodystrophy, vascular calcification

## Abstract

Osteoporosis and chronic kidney disease (CKD)–metabolic bone disease (MBD) (CKD-MBD) are increasingly recognized as overlapping conditions, particularly in the aging population. Declining renal function and skeletal fragility often coexist, because CKD-MBD may develop in a skeleton already compromised by preexisting osteoporosis. Adynamic bone, often resulting from excessive suppression of parathyroid hormone (PTH) and now a common form of renal osteodystrophy (ROD), may histologically resemble low-turnover osteoporosis; distinguishing between the 2 under light microscopy remains difficult, and reliable differentiation often depends on clinical context. Nevertheless, nephrologists and nonnephrologist bone specialists frequently work in parallel rather than in collaboration.This separation has contributed to persistent diagnostic gaps and fragmented management, especially in patients with advanced CKD. Advances in imaging, biochemical markers, and bone histomorphometry have improved insight into disease mechanisms; however, limitations in current diagnostic approaches remain. Osteoporosis therapies are frequently underused in CKD, despite growing evidence supporting efficacy and safety across a broader range of kidney function than previously assumed. Despite efforts to refine the definition of osteoporosis beyond bone mineral density (BMD) alone, clinical misclassification continues.Beyond skeletal health, vascular calcification (VC)—driven by disordered calcium–phosphate homeostasis—remains insufficiently prioritized in clinical decision-making, despite its strong association with cardiovascular morbidity and mortality in CKD. Emerging concepts, such as intermittent PTH administration, an established treatment in osteoporosis, illustrate the potential for interventions that may restore mineral balance and improve skeletal integrity in selected CKD populations. Whether such strategies can also favorably influence cardiovascular risk remains uncertain and warrant investigation. This integrated framework may improve interdisciplinary care.

Osteoporosis and CKD-MBD are increasingly recognized as intersecting conditions, especially within the context of an aging population.^1^ The growing recognition that declining renal function and osteoporosis frequently coexist in older adults has been underscored by recent publications from diverse authors and societies. To date, the management of osteoporosis in patients with CKD often occurs outside nephrology-led care. This is highlighted in a Lancet diabetes and endocrinology editorial or commentary,^2^ which, discussing the study by Bird *et al.*,^3^ noted that nephrologists were identified as prescribers of denosumab and oral bisphosphonates in only 2.3% and 2.6% of cases, respectively, among patients undergoing dialysis. Although this observation does not capture interdisciplinary discussions or shared decision-making, it nonetheless underscores the need for closer collaboration between nephrologists and bone specialists in the management of osteoporosis in CKD.

The cardiovascular complications of CKD, although the leading cause of mortality, continue to elude significant therapeutic advances and remain largely unprevented.

To begin to address this important deficit, the 25th WCO-IOF-ESCEO Congress, held in Rome from April 10 to 13, 2025, featured a symposium entitled “Osteoporosis and CKD-MBD under the Same Umbrella.” The symposium program was developed and coordinated by authors MP and PDM, both of whom were instrumental in its planning and execution, and was chaired by authors, NCH and DWD. There were 6 invited speakers; this report summarizes their presentations.

### Introduction - Osteoporosis Versus ROD –*Pieter Evenepoel*

In patients with CKD, optimal control of MBD is important not only to preserve cardiovascular health, but also to prevent debilitating skeletal complications. Patients with CKD experience a multifold increased fracture risk compared with age- and sex-matched controls and the risk of mortality following a hip fracture is substantially higher.^4^ Most alarming, the treatment gap, which is being already huge in postmenopausal women and men is even more pronounced in patients with CKD.^5^

Lack of evidence from randomized controlled trials, fear of complications, and incomplete understanding of the complex pathophysiology of bone fragility in CKD fuel therapeutic inertia. Originally, ROD has been referred to as osseous disorders simulating rickets, osteomalacia, or osteitis fibrosa cystica, but originating from chronic renal insufficiency.^6^ In 2006, Kidney Disease: Improving Global Outcomes launched the term CKD-MBD to describe a broader clinical syndrome that develops as a systemic disorder of mineral and bone metabolism because of CKD, which is manifested by abnormalities in bone and mineral metabolism and/or extraskeletal calcification.^7^ The Kidney Disease: Improving Global Outcomes recommended to use the term ROD exclusively to define alterations in bone morphology associated with CKD. At that time, osteoporosis and ROD were considered separate entities and mutually exclusive diagnoses. Consequently, nephrologists often deferred from osteoporosis management, whereas bone doctors avoided patients with CKD-MBD.^8^

Participants of the 2023 Madrid CKD-MBD Kidney Disease: Improving Global Outcomes Controversies Conference raised the concern that the term ROD may represent a roadblock to managing fracture risk because it fosters an overly PTH- and calcium-phosphate–centric approach to bone disease management. Because therapies focusing on these pathogenic drivers failed to meet expectations, the conference participants argued that treatment of renal bone disease should be recentered to the skeleton itself and that a change in terminology might facilitate this paradigm shift.^9^ The term CKD-associated osteoporosis was proposed to acknowledge and emphasize that ROD is a disorder of bone strength that increases fracture risk in the CKD setting.^9^^,^^10^ Although CKD-MBD biochemical abnormalities cannot be dissociated from the relevant clinical outcomes of bone loss and fractures, additional efforts are needed to better understand mechanisms by which uremic toxins, altered gut and immune systems, inflammation, and medications affect the CKD-associated osteoporosis phenotype.^9^

Rather than pointing to differences, clinicians should focus on similarities between the various types of osteoporosis. According to the updated conceptual framework, traditional and CKD-associated risk factors contribute to osteoporosis in patients with CKD. Diagnostic work-up and therapy are similar to what is proposed for other osteoporosis conditions but need to be individualized with knowledge of CKD. The bone phenotype can be primarily assessed using bone imaging and bone biomarkers. Treatment targets of PTH should be considered in the context of bone phenotype. This makes sense if we consider that a key feature of hyperparathyroidism in CKD is a reduced and variable skeletal response to PTH.^11^^,^^12^ Although, the metabolic bone component of CKD-associated osteoporosis is best evaluated using a bone biopsy, the key parameter of bone turnover, which has implications for treatment options, can be noninvasively assessed using bone turnover markers.^13^^,^^14^

Various consensus manuscripts have recently been published on the diagnosis and management of osteoporosis in patients with CKD G4-5D and may assist in clinical decision making.^15^, ^16^, ^17^, ^18^, ^19^ Failure to adhere to the “CKD-MBD first” principle in the therapeutic approach may explain the high incidence of hypocalcemia in denosumab-treated patients with advanced CKD.^3^^,^^20^^,^^21^ Adequate PTH (and bone turnover) suppression followed by bone targeting therapy is the most appropriate “sequential” therapy in patients with advanced CKD presenting with high bone turnover and osteoporosis. Benefits and risks of bone-targeting drugs should be balanced in a shared decision process, thus enabling off-label use of some antiresorptive drugs.^22^, ^23^, ^24^, ^25^ Expert advice from a multidisciplinary team, having access to bone histomorphometry and/or other advanced diagnostics as needed, may benefit complex cases.^26^

### Bone Histology - Osteoporosis Versus ROD – *David W. Dempster*

For over 50 years, histomorphometric analysis of the iliac crest bone biopsy has been one of the most powerful techniques to study bone metabolism.^27^ It owes its strength to the ability to produce high quality undecalcified sections of bone and the use of tetracycline antibiotics to provide dynamic assessment of bone formation rate, as well as disorders of mineralization. In the 1960s through the 1980s, the technique was used widely in the clinical assessment of patients with bone disease. Nowadays, with the advent of bone densitometry and biochemical markers of bone turnover, the technique is rarely used in a clinical setting, except in the management of patients with ROD. Indeed, bone biopsy is the gold standard to evaluate bone health in CKD. However, because of inherent limitations of the bone biopsy procedure and recent advances in biochemical markers, there is a growing consensus that bone biopsy as a diagnostic procedure should be reserved for complex cases, where bone turnover and mineralization status cannot be reliably determined by either biochemical markers or dual-energy x-ray absorptiometry (DXA).

The biopsy has taught us that ROD can present in several distinct forms: high bone turnover (osteitis fibrosa), osteomalacia, low turnover (adynamic bone), and mixed bone disease, commonly referred to under the umbrella of CKD-MBD. Concomitantly, patients with CKD-MBD also frequently present with osteoporosis. The iliac crest biopsy followed by histomorphometric analysis remains the most definitive way to differentiate among the different forms of CKD-MBD, including osteoporosis.

The turnover, mineralization, and volume (TMV) system to characterize the histological features of ROD is now widely accepted.^7^ As far as the concomitant presence of osteoporosis in CKD is concerned, volume is the key characteristic. The bone biopsy report should indicate whether cancellous bone volume is reduced and should include an assessment of trabecular architecture, because disrupted trabecular architecture could indicate osteoporosis in the presence of normal cancellous bone volume. Traditionally, bone histomorphometrists focus more on cancellous than cortical bone. However, when assessing volume in ROD, it is important to assess cortical thickness and porosity.

The bone biopsy will continue to be used clinically in complex cases of CKD-MBD. For example, when turnover or mineralization status cannot be reliably determined by biochemical markers. However, the invasive nature of the test, the labor-intensive nature of the analysis, and the world-wide shortage of bone histomorphometry laboratories and skilled histomorphometrists make routine evaluation of bone biopsies in CKD-MBD difficult. Advances in artificial intelligence and machine learning will help in this regard by automating measurements of certain histomorphometric parameters.^28^ Moreover, in the majority of clinical cases, a qualitative or semiquantitative assessment using the TMV classification system is often sufficient. Another limitation to the use of bone biopsy in difficult cases of CKD-MBD is the lack of good reference data. Obtaining normative histomorphometric data from healthy subjects is a challenge, especially as tetracycline labeling is necessary. Histomorphometric analysis is to a certain extent subjective and interlaboratory variability is high. Ideally, each histomorphometry laboratory should establish their own sex-, age-, and race-specific reference ranges. However, this is impractical. Establishment of reference ranges by averaging published values is an approach to this issue.^29^

In research studies, bone biopsy is invaluable for elucidating the effects of osteoporosis treatments on bone quality in CKD-MBD. Preliminary studies indicate that both antiresorptive and anabolic treatments can be effective in treating osteoporosis in CKD-MBD. Quadruple-labeled biopsies may be useful in this regard, allowing for a single biopsy to assess the effects of treatments on bone formation.^30^

### Diagnosis: Biochemistry and Imaging – Osteoporosis Versus ROD – *Thomas L. Nickolas*

Patients with CKD are at increased risk of skeletal complications, including fractures, because of a complex interplay of metabolic and structural bone abnormalities. Fracture incidence in patients with CKD is ≤ 4 times higher than in the general population, with associated increases in morbidity, mortality, and health care costs.^31^ Notably, patients aged < 45 years with CKD exhibit fracture rates similar to those aged > 65 years with normal renal function, reflecting a premature aging phenotype of the skeleton.^32^

Historically, bone disease in CKD has been classified under the umbrella of ROD, which primarily encompasses abnormalities in bone TMV.^4^ In contrast, osteoporosis is defined by low bone mass and the deterioration of bone microarchitecture, typically because of age-related or secondary causes, and diagnosed using BMD thresholds and fracture risk.^33^ However, this dichotomy is overly simplistic. Emerging understanding recognizes that CKD leads to a broader syndrome now often referred to as CKD-associated osteoporosis, which includes a confluence of features from both classical ROD and those commonly seen in osteoporosis.^34^ These overlapping conditions necessitate a nuanced diagnostic and therapeutic approach.

#### Biochemical Assessment

ROD is a disorder of bone turnover and biochemical analysis plays a role in determining its presence and severity. In ROD, abnormalities in calcium, phosphate, PTH, and vitamin D metabolism characterize the presence of the disorder in bone.^35^ Elevated PTH, altered phosphate handling, and deficient or dysregulated vitamin D metabolites are biochemical hallmarks of ROD. Bone turnover markers that are not renally cleared, such as bone-specific alkaline phosphatase, procollagen type I N-terminal propeptide, and tartrate-resistant acid phosphatase 5b, can help assess remodeling status and differentiate between high- and low-turnover bone disease in CKD.^17^

In contrast, primary osteoporosis is primarily a disorder of bone mass and microarchitecture. Generally, it presents with normal mineral metabolism and PTH levels. The diagnosis is either by assessment of BMD using DXA or history of fragility fracture.^33^ Bone turnover markers may be elevated or reduced depending on the pathophysiology but are not typically used in isolation for diagnosis.

Thus, though both ROD and osteoporosis conditions involve changes in bone turnover, the biochemical profiles differ significantly in origin and interpretation. However, changes in bone mass and microarchitecture may be similar between the 2 disorders.

#### Imaging

Imaging assessments for bone disease in CKD have traditionally lagged behind that in osteoporosis. DXA is the clinical standard tool for evaluating BMD and assessing fracture risk in both the general and the CKD population. It is important to note that DXA is unable to determine bone remodeling in any population of patients with bone disorders.

DXA cannot be used to diagnose the underlying cause from any etiology. Therefore, though a low T-score may reflect increased fracture risk, it does not inform the underlying pathophysiology of bone disease in patients with CKD. Other imaging modalities such as high-resolution peripheral quantitative computed tomography and trabecular bone score offer insights into bone microarchitecture. Although trabecular bone score is becoming widely used, high-resolution peripheral quantitative computed tomography remains a research tool.^36^

In patients with CKD, ROD and osteoporosis do not develop and progress in a vacuum but in parallel and concomitantly, with risk factors that are both shared (i.e., age, sex, hypogonadism) and unique to the uremic environment.^37^ Thus, for practical and treatment purposes, it may be impossible to determine the unique contributions of each condition to the clinical phenotype. Nonetheless, treatment strategies should reflect this clinically relevant challenge. A personalized, sequential, and comprehensive strategy is essential—beginning with correction of mineral metabolism disturbances, followed by fracture risk stratification and targeted treatment. Recognizing the shared and distinct elements of osteoporosis and ROD is crucial for improving outcomes in this high-risk population.

### VC in CKD-MBD and associated Osteoporosis – *Ziad A. Massy*

CKD affects approximately 10% of the global population and is commonly associated with both VC and skeletal fragility. These complications are hallmarks of CKD-MBD, a systemic disorder that affects mineral metabolism, bone turnover, and extraskeletal calcification.^9^ In older individuals with CKD, age- and sex-dependent osteoporosis often coexists, further complicating the diagnosis and management of bone disease and its vascular implications.^38^ VC, particularly in the form of arterial and valvular calcification, is strongly associated with increased cardiovascular and all-cause mortality.^39^^,^^40^ In patients with CKD, VC is highly prevalent and progresses more rapidly than in the general population. It is driven by a complex interplay of metabolic dysregulations, including hyperphosphatemia, hyperparathyroidism, inflammation, oxidative stress, and accumulation of uremic toxins. These factors accelerate phenotypic transitions of vascular smooth muscle cells into osteoblast-like cells, facilitating calcification in the arterial media and cardiac valves. Clinically, VC is linked to arterial stiffness, increased pulse pressure, left ventricular hypertrophy, and valvular stenosis or regurgitation.

In contrast, though VC in individuals with primary osteoporosis is not a defining feature of the disease, it is increasingly recognized as a parallel pathology. Epidemiological studies have demonstrated that postmenopausal women and elderly men with low BMD have a higher prevalence of aortic calcification and subclinical measures of atherosclerosis. Although the mechanisms are less clearly defined, shared risk factors, including aging, estrogen deficiency, chronic inflammation, and oxidative stress, may contribute to a common soil for both osteoporosis and vascular disease.

Despite these differences, there is a growing interest in the bone-vascular axis—specifically, the concept of "calcification paradox," where patients lose mineral from bone while gaining mineral in vascular tissue. Several studies have reported inverse associations between VC scores and BMD.^10^ However, causal relationships remain elusive. The proposed mechanisms include the following: (i) endocrine and paracrine actions of bone-derived factors (e.g., osteocalcin, FGF-23, sclerostin) on vascular tissues^41^; (ii) shared progenitor cells and signaling pathways; (iii) reduced skeletal blood flow and impaired bone perfusion^42^; and (iv) common systemic insults such as uremic toxins, inflammation, and oxidative stress.^43^ CKD presents a unique model of accelerated aging, in which these pathologies are magnified and more tightly interwoven.^38^ The convergence of osteoporosis and VC is particularly evident in elderly patients with CKD, where distinguishing between primary and secondary bone disease becomes clinically relevant. Moreover, treatment decisions, especially those involving antiresorptive or anabolic therapies, must consider potential effects on both the skeleton and the vasculature.

Importantly, some common risk factors are modifiable and could be targeted for dual-benefit interventions. For example, the control of serum phosphate, reduction of chronic inflammation, and mitigation of oxidative stress are all strategies that may improve both bone and vascular outcomes in CKD.

Although CKD-MBD and osteoporosis differ in etiology and clinical presentation, they share overlapping pathways that link bone fragility to VC. Understanding these interconnections is essential for developing more effective, integrated approaches to the prevention and treatment of skeletal and cardiovascular complications in CKD and in the general aging population.

### Current Therapeutic Options - Osteoporosis and ROD – *Sandro Mazzaferro*

The concept of CKD-associated osteoporosis has been recently introduced to underscore the dual burden of skeletal fragility in patients with CKD.^9^ These patients face fractures because of both classical osteoporosis risk factors and the distinct metabolic bone disorders associated with ROD. Although fracture rates are alarmingly high across this population and associated with significant morbidity and mortality,^44^ the pathogenesis is heterogeneous, varying with CKD duration, glomerular filtration rate, and severity of secondary hyperparathyroidism (SHPT). Therefore, tailoring therapeutic strategies requires a nuanced understanding of both conditions.^9^

A common ground in managing both osteoporosis and ROD is the emphasis on lifestyle modification. Smoking cessation; limited alcohol consumption; optimal nutrition, particularly calcium balance; and regular weight-bearing exercise are crucial across all patient groups. Fall risk assessments and minimization, especially through careful review of concurrent medications, are similarly essential.^45^

SHPT management is another pivotal target in both conditions. Ensuring a balanced calcium intake (800–1000 mg/d, not exceeding 1500 mg/d) is critical in renal patients to avoid both deficiency and excess, which could exacerbate bone disease or VC.^12^ Vitamin D status must be optimized, with cholecalciferol or calcifediol supplements being appropriate in early CKD stages, whereas activated forms (e.g., calcitriol or paricalcitol) may be reserved for more advanced disease, albeit cautiously because of the risk of hypercalcemia.^46^

A clear divergence appears when considering bone-directed pharmacotherapies, particularly because of renal excretion and altered drug metabolism.

• Bisphosphonates, the cornerstone of osteoporosis therapy in the general population, have important limitations in patients with CKD, particularly in advanced stages (G4–G5). Reduced renal clearance, altered pharmacokinetics with prolonged skeletal retention, and the potential risk of exacerbating low bone turnover or adynamic bone warrant caution when bone turnover status is uncertain.^22^^,^^24^ Although bisphosphonates improve BMD, robust evidence for fracture reduction in patients with advanced CKD or on dialysis is lacking; and current guidelines generally advise against their use when estimated glomerular filtration rate is < 35 ml/min.^47^ These considerations do not preclude their use in selected patients with preserved renal function or well-characterized bone turnover, but underscore the need for individualized risk–benefit assessment and close collaboration between nephrologists and bone specialists when antiresorptive therapy is considered in CKD.
• Denosumab, an anti-RANKL monoclonal antibody, offers theoretical advantages because it is not renally excreted.^48^ It is effective in postmenopausal osteoporosis and can be used in early CKD.^49^ However, in advanced CKD, it carries risks of hypocalcemia. Furthermore, in any population in which it is used, there is the risk of rebound-associated bone loss and spine fractures upon discontinuation.^50^ Sequential therapy in the general population suggests that the use of a high potent bisphosphonate can mitigate the risk; although, primary data in patients with CKD are lacking.
• Raloxifene, a selective estrogen receptor modulator, has been used in postmenopausal women on dialysis with some success in improving BMD, though its role remains exploratory, based primarily on observational data.

#### Management of SHPT

• Calcium*-*sensing receptor agonists (e.g., cinacalcet), used to manage SHPT, are effective in dialysis patients but unsuitable in earlier CKD because of their tendency to increase phosphate retention and lower serum calcium excessively.^51^ They have not been shown to prevent fractures in clinical trials.

Anabolic agents represent a promising frontier, especially relevant in CKD-MBD, where bone formation is often impaired. However, their use is complicated by the coexistence of SHPT.

• Teriparatide, a PTH^1^, ^2^, ^3^, ^4^, ^5^, ^6^, ^7^, ^8^, ^9^, ^10^, ^11^, ^12^, ^13^, ^14^, ^15^, ^16^, ^17^, ^18^, ^19^, ^20^, ^21^, ^22^, ^23^, ^24^, ^25^, ^26^, ^27^, ^28^, ^29^, ^30^, ^31^, ^32^, ^33^, ^34^ analog, has shown efficacy in osteoporosis, including mild CKD stages.^52^ Its paradoxical role in patients with SHPT raises concerns, but studies in dialysis patients suggest BMD improvements, though data on fracture reduction are lacking.
• Abaloparatide, a PTHrP analog with similar anabolic properties, has been used in patients with osteoporosis with mild CKD^53^; however, evidence in advanced stages or dialysis is scarce.
• Romosozumab, an antisclerostin antibody with combined anabolic and antiresorptive effects, has shown BMD gains and changes in bone turnover markers in observational studies involving dialysis patients.^54^ However, its use is limited by concerns regarding cardiovascular safety, including a US Food and Drug Administration black-box warning for increased risk of myocardial infarction and stroke, as well as by the risk of hypocalcemia, considerations of particularly relevant in CKD-MBD.^55^

Despite the availability of these agents, there is a lack of high-quality randomized controlled trials specifically targeting patients with advanced CKD. Most data are derived from postmenopausal osteoporosis populations, with limited applicability to the broader CKD cohort that includes men and other age ranges. A recent systematic review underscored this gap, concluding that though some therapies may modestly reduce vertebral fractures or improve bone strength, the evidence is limited and inconclusive.^56^^,^^57^

Although nonpharmacologic strategies and SHPT management form the backbone of treatment for both osteoporosis and ROD across all CKD stages, pharmacologic options must be carefully selected based on the stage of kidney disease and underlying bone pathology. Anabolic therapies hold promise for addressing the bone formation deficits of CKD-MBD but require cautious implementation and further validation. Until robust clinical trials provide clearer guidance, individualized therapy grounded in a thorough metabolic and clinical assessment remains essential.

### Intermittent PTH Administration in the Management of CKD-MBD – *Michael Pazianas*

Calcium and phosphate are essential for human physiology, tightly regulated by a hormonal network involving the parathyroids, bone, kidneys, and gastrointestinal tract. Key regulators, namely, PTH, FGF-23, Klotho, and 1,25-dihydroxyvitamin D, maintain mineral balance, with the kidneys acting as a critical checkpoint that can modulate calcium and phosphate excretion independently.

In CKD, declining renal function disrupts this equilibrium, giving rise to CKD-MBD. For decades, the “trade-off” hypothesis, centered on phosphate retention, and the knowledge that increased PTH and SHPT occur long before increases in serum phosphate levels are evident, guided our understanding of this syndrome. PTH suppression, therefore, was the primary therapeutic goal, based on the premise that controlling SHPT would prevent bone complications (the PTH-centric model). Strategies to suppress PTH, including calcium supplements and potent vitamin D analogs, initially succeeded in reducing SHPT incidence, but at a cost—the widespread emergence of adynamic bone.

The discovery of FGF-23 has fundamentally reshaped our understanding of CKD-MBD. This phosphaturic hormone increases early in the course of CKD—well before detectable changes in serum phosphate or PTH—suppressing 1,25-dihydroxyvitamin D production and initially dampening PTH secretion. These observations do not challenge the PTH-centric model itself but rather the way we interpret data derived from it. Specifically, they highlight what appears to be a delayed or "lagged" PTH response (PTH hysteresis) in the early stages of CKD-MBD. Concurrent calcium retention,^1^ along with phosphate retention, may adequately account for this PTH lag, allowing FGF-23 to dominate the early pathophysiological landscape.

Given this, we propose that intermittent PTH administration early in CKD may restore balance. Supplementing PTH intermittently could enhance phosphate excretion, reduce the drive for FGF-23 elevation, maintain 1,25-dihydroxyvitamin D levels, and prevent the maladaptive hormonal responses underlying CKD-MBD. Crucially, intermittent PTH—distinct from the chronically elevated levels in SHPT—could offer anabolic skeletal benefits while preserving mineral homeostasis.^1^

This strategy may mitigate VC, a key contributor to cardiovascular mortality in CKD. By improving calcium-phosphate dynamics and preventing the FGF-23 surge, early PTH therapy has the potential to alter the natural history of CKD-MBD and its cardiovascular consequences.^1^

Intermittent administration of PTH (teriparatide) and its analogue abaloparatide, an established treatment for osteoporosis, has shown promising effects on calcium and phosphate homeostasis and bone metabolism in CKD-MBD.

In mice or individuals with normal renal function PTH administration had significant effects on FGF-23 levels. A single subcutaneous injection of PTH^1^, ^2^, ^3^, ^4^, ^5^, ^6^, ^7^, ^8^, ^9^, ^10^, ^11^, ^12^, ^13^, ^14^, ^15^, ^16^, ^17^, ^18^, ^19^, ^20^, ^21^, ^22^, ^23^, ^24^, ^25^, ^26^, ^27^, ^28^, ^29^, ^30^, ^31^, ^32^, ^33^, ^34^ in mice increased circulating cFGF-23 levels, followed by proteolytic cleavage and a decline in intact FGF-23 to approximately 60% within 6 hours.^58^ Similarly, a 6-hour i.v. infusion of PTH in healthy humans led to decreased FGF-23 levels and reduced serum phosphate, whereas serum calcium remained inside the normocalcemic range.^59^ In postmenopausal women treated for osteoporosis over 18 months, long term intermittent PTH was associated with a reduction in endogenous PTH, potentially lowering the risk of parathyroid hyperplasia and SHPT, and an increase in FGF-23 levels.^60^ However, these outcomes may have been influenced by sample timing, because another study in mice found that 20 days of daily PTH decreased iFGF-23 while increasing 1,25(OH)_2_D.^61^

In CKD-MBD animal models, intermittent teriparatide increased bone formation despite the presence of SHPT, with minimal changes in serum calcium or phosphate, and was accompanied by reductions in both FGF-23 and iPTH levels.^62^ When administered for 4 weeks, it not only prevented bone loss but was also associated with reduced VC.^63^ Although these findings are hypothesis-generating, comparable data in humans, with respect to vascular or cardiovascular end points, are currently lacking.

Human data supports the safety and efficacy of teriparatide and abaloparatide in patients with mild to moderate renal impairment. In the Fracture Prevention Trial, teriparatide was generally well-tolerated, with only transient, mild hypercalcemia and no serious adverse events.^52^ Likewise, the ACTIVE trial demonstrated that abaloparatide remained effective and safe across varying degrees of renal function.^53^ Postmarketing surveillance from Japan confirmed teriparatide’s tolerability in advanced CKD, with few and nonserious adverse drug reactions.^64^ In patients with stage 4 and 5 CKD, limited off-label use of teriparatide for adynamic bone has shown promising outcomes.

Intermittent PTH or analogue administration may be particularly beneficial in patients with early-stage CKD with rising FGF-23. Timely intervention at the onset of rising FGF-23, using carefully titrated PTH regimens, may help restore mineral homeostasis and prevent downstream complications. With further validation, this strategy could offer a novel, preventive approach to CKD-MBD management.

### Rethinking Osteoporosis and CKD-MBD

CKD can contribute to the development of osteoporosis through multiple CKD-dependent mechanisms, including abnormalities in mineral metabolism, hormonal dysregulation, inflammation, gut dysbiosis, and altered bone remodeling and mineralization. Conversely, CKD-MBD may develop on a preexisting osteoporotic skeletal framework, particularly in aging individuals, where both CKD and osteoporosis are more prevalent.

Despite the overlapping risk factors and clinical manifestations, osteoporosis is not formally classified among the traditional forms of ROD. Historically, in the 1980s, efforts to prevent SHPT through aggressive suppression of PTH led to the emergence of a distinct form of bone disease, adynamic bone. Histologically, adynamic bone is defined by low bone turnover, reduced osteoblast and osteoclast activity, and diminished or absent bone formation; features that resemble low bone turnover osteoporosis in many ways. However, osteoporosis itself lacks a formal histological definition, and interestingly, there is no definitive method to distinguish the 2 conditions under light microscopy.^65^

The term osteoporosis is often used interchangeably, and inaccurately, with any condition involving low BMD or reduced bone strength. This misconception can be traced back to 1994, when the World Health Organization defined osteoporosis solely based on BMD thresholds.^66^ Although the National Institutes of Health refined this definition in 2000 to emphasize that bone strength, encompassing both BMD and bone quality, is the true determinant of fracture risk,^67^ the misunderstanding persists. As a result, low BMD is still widely perceived as a hallmark of osteoporosis, overlooking the fact that other conditions, such as osteomalacia, can present with low BMD. These disorders differ fundamentally in their pathophysiology and therefore require distinctly different diagnostic and therapeutic strategies.

The Kidney Disease: Improving Global Outcomes guidelines, introduced in 2006, proposed the TMV classification system—assessing bone turnover, mineralization, and volume—to evaluate renal bone disease, but made no mention of osteoporosis in this context. Even in the 2009 update, the guidelines maintained this distinction. Around the same time, terms such as “CKD-induced osteoporosis” or “CKD-MBD–associated osteoporosis” began to emerge in the literature, reflecting an evolving recognition of the overlap between the 2 conditions.

Regardless of whether adynamic bone and osteoporosis are part of a shared spectrum or entirely distinct entities, both are increasingly treated with intermittent PTH therapy—an anabolic intervention aimed at restoring bone turnover and improving bone mass.

This convergence in therapeutic approach highlights the need for a more unified framework that recognizes the interplay between CKD-MBD and osteoporosis. Ultimately, rather than viewing osteoporosis and CKD-MBD as competing frameworks, it may be more productive to recognize them as different expressions of compromised skeletal health. Aligning the 2 under a common clinical and conceptual umbrella enables a more unified approach to diagnosis, research, and therapy—improving the care of a vulnerable and often overlooked patient population (Figure 1 and Table 1).

**Figure 1:**
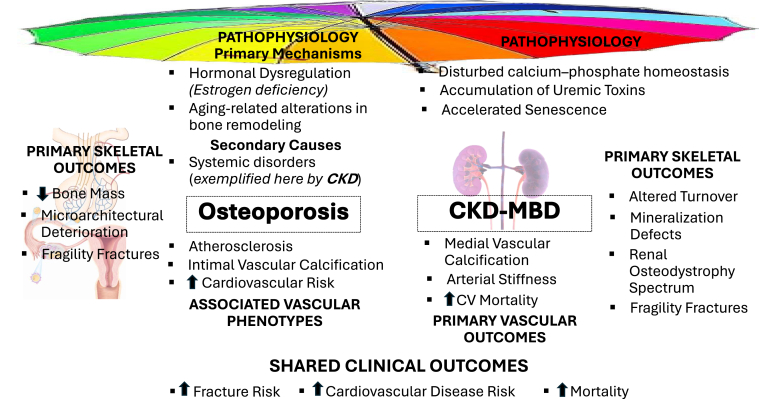
Osteoporosis and CKD-MBD are shown as biologically distinct disorders—hormonally driven and mineral-homeostasis–driven, respectively—with convergence at skeletal fragility and cardiovascular risk through distinct mechanistic pathways, including disease-specific metabolic stress and accelerated cellular aging. CKD-MBD, chronic kidney disease–metabolic bone disease; CV, cardiovascular.

**Table 1:** Comparison of osteoporosis and CKD-MBD across key clinical domains, highlighting differences in biochemical assessment, bone turnover patterns, diagnostic tools, vascular involvement, and therapeutic decision-making

Clinical domain	Osteoporosis	CKD-MBD
Biochemistry (routine clinical testing)	Serum Ca, PTH, and Vit D are often normal	Altered Ca–Pi–PTH–FGF23 and Vitamin D axis
Imaging	DXA-derived BMD	DXA-derived BMD for fracture risk assessment in CKD; adjunctive imaging (e.g., plain radiography, and HR-pQCT)
Bone biopsy (diagnostic yield)	Rarely performed; typically shows reduced bone volume with variable turnover and impaired microarchitecture	Reference standard when indicated; High, low, or mixed turnover; mineralization abnormalities
Vascular calcification	Common comorbidity	Pathophysiologically linked to mineral dysregulation
Therapeutic Approach	Antiresorptives/Osteoanabolics	Personalized treatment based on CKD-MBD severity and type, fracture risk classification, clinical risk factors, and turnover status

BMD, bone mineral density; Ca, calcium; CKD-MBD, chronic kidney disease–metabolic bone disease; DXA, dual energy x-ray absorptiometry; FGF23, fibroblast growth factor 23; HR-pQCT, high-resolution peripheral quantitative computed tomography; Pi, inorganic phosphate; PTH, parathyroid hormone; Vit D, vitamin D.

The broader and most significant gain from bringing “osteoporosis and CKD-MBD under the same umbrella” would be to bridge the gap between renal and nonrenal bone specialists, fostering collaboration and ensuring patients receive appropriate care. Currently, renal clinicians often defer osteoporosis management, whereas nonrenal specialists are hesitant to treat patients with CKD with osteoporosis. In addition to improving skeletal outcomes, this integrated approach could benefit the often-overlooked vascular component of CKD-MBD. By aligning efforts and therapeutic strategies, we may gain deeper insights into the shared mechanisms linking bone and vascular health—particularly the relationship between osteoporosis, VC, and cardiovascular morbidity and mortality—thereby offering a more holistic and potentially transformative way to manage this complex disease spectrum.

## Disclosure

DWD reports consultancy, grant support, and speaker fees from Amgen, Inc. and Radius Health. PE reports grants from Vifor CSL and Sanofi, participation on speaker bureaus for UCB, and an advisory board role at Vifor CSL, all unrelated to the submitted work. TLN reports receiving honoraria and support from Amgen (speaker, grant support, and advisory board) and Intas Pharmaceuticals (speaker). ZAM reports grants for CKD-REIN and other research projects from Amgen, Baxter, Fresenius Medical Care, GlaxoSmithKline, Merck Sharp & Dohme-Chibret, Sanofi-Genzyme, Lilly, Otsuka, AstraZeneca, Vifor, and the French government; and fees and grants to charities from AstraZeneca and Boehringer Ingelheim. NCH reports receiving consultancy, lecture fees, honoraria, and/or grant funding from Alliance for Better Bone Health, Amgen, MSD, Eli Lilly, Radius Health, Servier, Echolight, Shire, UCB, Consilient Healthcare, Kyowa Kirin, Theramex, and Internis Pharma. All the other authors declared no competing interests.
